# Impact of a protein-based assay that predicts prostate cancer aggressiveness on urologists’ recommendations for active treatment or active surveillance: a randomized clinical utility trial

**DOI:** 10.1186/s12894-017-0243-1

**Published:** 2017-07-03

**Authors:** John W. Peabody, Lisa M. DeMaria, Diana Tamondong-Lachica, Jhiedon Florentino, M. Czarina Acelajado, Othman Ouenes, Jerome P. Richie, Trever Burgon

**Affiliations:** 1 QURE Healthcare, 450 Pacific Ave, Suite 200, San Francisco, CA USA; 2 University of California, San Francisco, 500 Beale Street, San Francisco, CA USA; 3 Metamark Genetics, 245 First Street, 10th Floor, Cambridge, MA USA

**Keywords:** Proteomic biomarker, Protein-based assay, Gleason score, Active surveillance, Active treatment, Simulated patients, Evidence-based treatment

## Abstract

**Background:**

Of the more than 1.1 million men diagnosed worldwide annually with prostate cancer, the majority have indolent tumors. Distinguishing between aggressive and indolent cancer is an important clinical challenge. The current approaches for assessing tumor aggressiveness are recognized as insufficient. A validated protein-based assay has been shown to predict tumor aggressiveness from prostate biopsy. The main objective of this study was to measure the clinical utility of this new assay in the management of early-stage prostate cancer.

**Methods:**

One hundred twenty nine board-certified urologists were asked to participate in a randomized, two-arm experiment. We collected data over 2 rounds using simulated clinical cases administered via an online platform. The cases were all newly diagnosed Gleason 3 + 3 or 3 + 4 prostate camcer patients. Urologists in the intervention arm received a 15-min webinar on this protein-based assay and given assay test results for their simulated patients in round 2. Each case had a preferred recommendation of either active surveillance or active treatment. The measured outcome was rate of preferred recommendation, defined as urologists who recommended the proper treatment course. Analyses were done using difference-in-difference estimations.

**Results:**

Using multinomial logistical regression, urologists who were given the assay results were significantly more likely to choose the preferred recommendation (active surveillance or active treatment) compared to controls (*p* = 0.004). These urologists were also significantly more likely to involve their patients in the treatment decision compared to controls (*p* = 0.001).

**Conclusions:**

By providing additional information to inform the physician’s treatment plan, a protein-based assay shows demonstrable clinical utility confirmed through a rigorous randomized controlled study design and regression analyses to test for effects.

## Background

Worldwide, prostate cancer is the most commonly diagnosed solid organ tumor and the second deadliest, with more than 1.1 million new cases in 2012 [1]. Depending on emphasis of early detection and/or treatment, wide variation exists in mortality rates in various countries [2]. Over recent decades, however, widespread use of prostate-specific antigen (PSA) testing has led to a pronounced shift toward identification of early-stage tumors, many of which are likely indolent and ultimately present little or no risk to the patient [3]. Simultaneously, treatment advances in robotic surgery and advanced radiation therapy have opened up additional opportunities to aggressively treat localized tumors, which may subject some patients to unnecessary treatment and the risks. These advances are key drivers in the rising cost of prostate cancer treatment [4]. Because of this, active surveillance is increasingly being recommended as a treatment option [5].

Distinguishing between aggressive and indolent cancer, and delivering the appropriate level of care to each group, is thus an important clinical and economic challenge. Active surveillance (AS) protocols, which emphasize identification of low-risk patients and monitoring of the tumor in lieu of aggressive treatment, have been incorporated into U.S. and European guidelines [6, 7], are linked with the highest quality-adjusted life expectancy [8], but appear to be underutilized [9], with significant variation in adoption that cannot be explained by clinical characteristics [10]. The decision to pursue AS is a complex process where clinical risk stratification, physician recommendation, and patient preference all play roles [11–14]. In a recent review [14], the researchers noted that while a uniform approach to AS would be appealing, “current diagnostic and prognostic tools lack the precision needed to reliably monitor men… [who have] varying risks and preferences.”

Evaluation of appropriate use of AS and active treatment (AT) is plagued by subtle differences in patient presentation *and* differences in clinical treatment decisions. If, however, patient level variability could be controlled, the (expected high level of) variability in clinical decision making between AS and AT, defined as providing a clinical intervention [11, 15], could be understood.

Given the wide variation in prostate cancer treatment decisions, a key question is whether better diagnostic tests for assessing tumor aggressiveness leads to better treatment decisions and better clinical utility. Given the preponderance of low risk patients, diagnostics that increase confidence in risk assessment will also likely result in reduced health care spending. A new quantitative immunofluorescent protein-based assay (ProMark) that predicts tumor aggressiveness at biopsy has been described for patients with Gleason grades 3 + 3 or 3 + 4 prostate cancer, wherein aggressiveness is difficult to distinguish using existing clinical and pathological parameters [16].

The objective of this study is to measure the variability of treatment decisions of AT or AS and determine the impact of adding a protein-based risk assessment assay to current standard of care risk classification approaches in the management of early-stage prostate cancer. To overcome patient-level (case-mix) variability, this study uses validated case simulations of early stage prostate cancer, among a large cohort of urologists.

## Methods

### Design

A “before-and-after” design was utilized in a longitudinal randomized controlled study of board-certified urologists practicing in the U.S. Urologists were asked to care for online simulated patients with underlying but undiagnosed prostate cancer, using Clinical Performance and Value (CPV®) vignettes via web-based interactive ‘patient visits.’ All patients had a Gleason Score of 3 + 3 = 6 or 3 + 4 = 7. CPVs are simulated cases wherein clinicians are asked to interview and examine patients and order investigational studies including biopsies and blood tests. Clinicians taking care of the simulated patient are provided with responses to all history and physical items and results for any tests or procedures they choose to order. They are asked to diagnose the patient and make a treatment plan based on their investigations.

Physicians were randomized into control or intervention study arms and completed three vignettes at baseline (Round 1) and another three 6 to 8 weeks later (Round 2). In Round 1, no urologist received any information about the protein-based assay test, and none of the vignettes included these results. Between Rounds 1 and 2, intervention group urologists were introduced to the protein-based assay via a 15-min informational video. Intervention participants were then provided with these results for each Round 2 vignette. Protein-based assay information and results were not made available to the control group. The study design was approved by Chesapeake IRB (Columbia, MD). All participants provided written consent to participate.

### The protein-based assay

The protein-based assay is a novel 8-biomarker proteomic test, using quantitative multiplex immunofluorescence to measure protein levels on prostate biopsy tissue. The assay produces a prognostic risk score scaled from 0 to 100 and stratifies patients into low (0–33), intermediate (34–60) or high risk (61–100). These scores independently predict final disease pathology and assess disease aggressiveness [16]. This test supplements current prostate cancer risk assessment methods, especially in cases where existing tools do not clearly delineate the appropriateness of AS or AT.

### Eligibility and selection of physicians

**Fig. 1:**
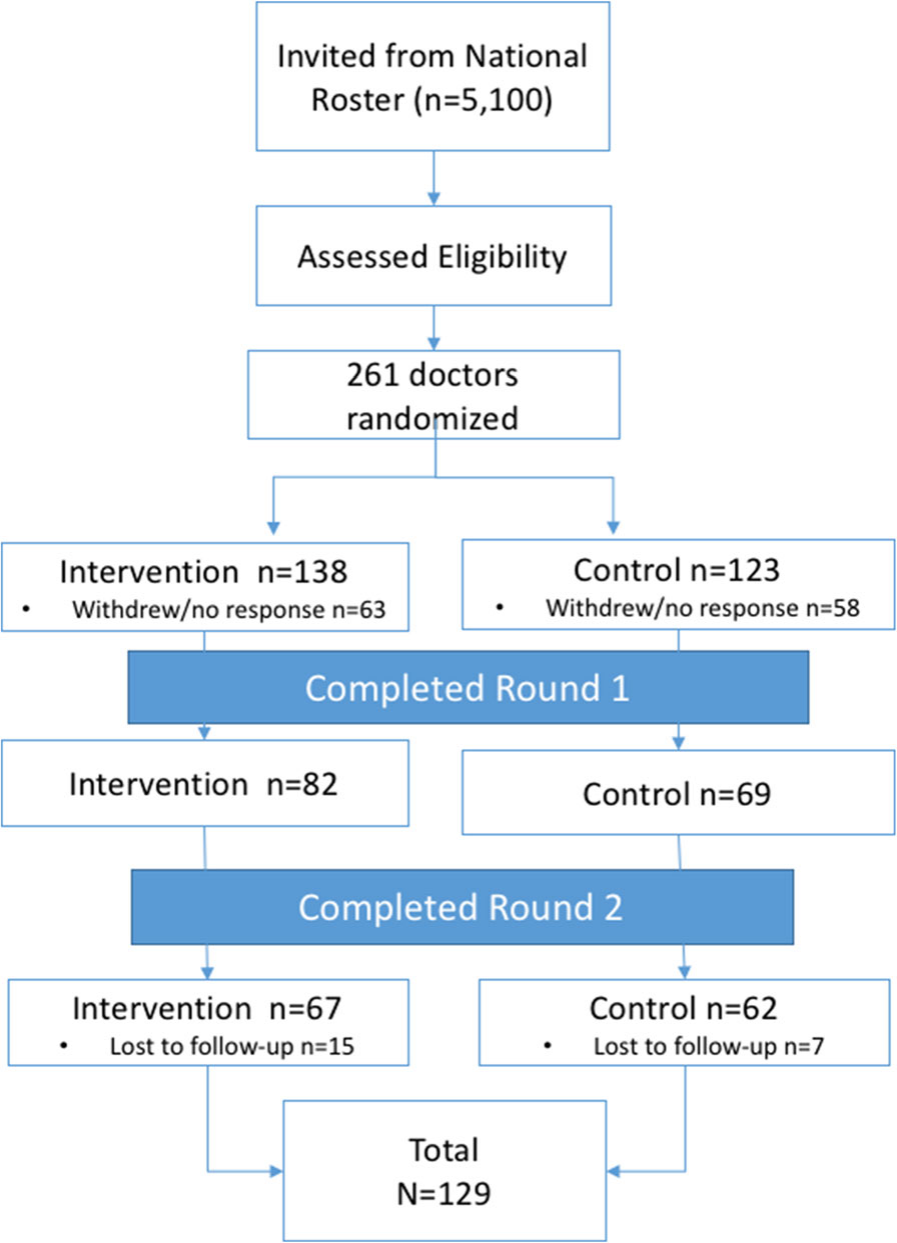
Flowchart of Sample Selection

Physicians had to (1) be currently practicing board-certified urologists, (2) have practiced (as a board-certified urologist) for greater than 2 or less than 30 years, (3) be English-speaking, (4) practice in a community/non-academic setting, (5) have ≥50 prostate cancer patients under care annually, (6) have Internet access, (7) have no prior experience with the protein-based assay test and (8) provide consent to participate in the study. Potential participants were contacted from a list of approximately 5100 practicing urologists who were randomly selected and invited to participate. Eligibility requirements, screening tools and study information presented during recruitment were identical for both groups. Eligible urologists were invited to participate in the study and 261 initially consented (Fig. 1). These were randomized into one of two arms: 138 in intervention and 123 in control. Of those initially randomized, 82 intervention and 69 control physicians participated at baseline, and 67 intervention and 62 control physicians completed the second round of CPVs. Statistical analysis yielded no significant differences between the physicians recruited in either of the two strategies nor among those who dropped out of the study or were lost to follow up.

### Clinical Performance and value® vignettes

Treatment choice and treatment utility were measured at baseline (Round 1) and after 6–8 weeks (Round 2) using Clinical Performance and Value (CPV®) vignettes. CPVs are a validated means for assessing differences in clinical practice and inherent variation in care, independent of case-mix [17, 18]. All providers care for the same patients and patient types, eliminating patient variability or observed and unobserved patient heterogeneity from the analysis and allowing for whether a test changes clinical practice; both of which are difficult to completely overcome through chart review analysis.

The CPVs were designed around the evidence-base with clear appropriate recommendations for treatment courses. The vignettes simulated clinical encounters involving men presenting with suspected early stage prostate cancer, verifiable by ordering a biopsy. In each vignette, the urologists were asked to ‘care for the patient’ by answering open-ended questions regarding the clinical care they would provide. Responses were requested and scored in: taking a medical history, performing a physical examination, ordering appropriate diagnostic tests (including laboratory tests, imaging studies and procedures), determining a diagnosis, and outlining a treatment plan (i.e., AS or AT). Explicit scoring criteria were established prior to study administration and were derived from a literature review, National Comprehensive Cancer Network guidelines, and expert opinion. Completed vignettes were scored as a percentage of physician answers matching these evidence-based criteria.

Three groups of three (9 total) CPV vignettes, representing typical cases for practicing urologists, were written to evaluate the variability of AS versus AT and the impact of protein-based assay test results on patient management (Table 1). All nine cases were undiagnosed Gleason 3 + 3 and 3 + 4 prostate cancer patients, Stage T1c or T2a, specified by their activity level, an elevanted PSA and risk categories such that each case had a preferable treatment course that was either AS or AT (Table 2). The primary outcome measure was the appropriate recommendation according to the individual case.

**Table 1:** List of Case Types, 3 CPV Cases within Each Type

Case Type	Standard	Standard + Protein-based Assay
A	Evidence-based treatment^a^	Assay confirms treatment
B	Evidence-based treatment^a^	Assay recommends switch (e.g., AS to AT)
C	Ambiguous treatment course	Assay resolve ambiguity

^a^Either AS or AT (depending on individual case) and based on age, PSA, Gleason score, etc

**Table 2:** CPV Case Details

CPV Case	Presenting History	Stage	Gleason	Cores	PSA	NCCN Risk Category	PBA score	PBA Risk Category	Preferred Option
1	Active 60 year old M with increasing urinary frequency	T1c	3 + 3	2_12 (<50%)	<10	Very Low	15	Low	AS
2	Sedentary 78 year old M with hematuria	T1c	3 + 3	4_12 (<50%)	7.1	Very Low	15	Low	AS
3	Moderately active 73 year old M with urinary frequency and hesitancy	T2a	3 + 4	3_12 (20% tumor in 4 s)	14	Intermediate	17	Low	AS
4	Quite active 57 year old M with erectile dysfunction and recent prostatitis	T2a	3 + 4	7_12	21	High	17	Low	AS
5	Moderately active 55 year old M with rising serum PSA levels	T1c	3 + 3	4_12	9.8	Very Low	35	Intermediate	AS
6	63 year old M, no longer active due to knee osteoarthritis, with suspicious digital rectal examination	T1c	3 + 3	6_12	8.9	Low	60	Intermediate	AT
7	Active 62 year old M with gross hematuria	T1c	3 + 4	3_12 (10% showing)	10.4	Intermediate	20	Low	AS
8	Lightly active 75 year old M seen for follow-up of suspicious nodularity on prostate	T2a	3 + 4	6_12	8.7	Intermediate	77	High	AT
9	Moderately active77 year old M seen in referral for nodule on prostate and high serum PSA	T2a	3 + 4	6_12	22.1	High	77	High	AT

*PBA* protein-based assay

### The intervention

Prior to Round 2, the intervention group was given information about the protein-based assay via a 15-min informational video that provided an overview of the test and interpretation of the scores. 87.5% of the intervention group watched the video webinar. The intervention group then received hypothetical protein-based assay scores and disease aggressiveness risk estimates for each patient case in the Round 2 vignettes.

### Analysis

The analysis determined how frequently did, urologists caring for Gleason 3 + 3 and 3 + 4 prostate cancer patients, recommend the preferred treatment pathway, and did this increase with the introduction of the protein-based assay? The preferred treatment pathway, defined on a case-by-case basis, was either AS or AT. Physician treatment was classified into four mutually exclusive categories: preferred treatment for the case; suboptimal treatment, defined as recommending AS when the case presentation and clinical guidelines indicated AT or recommending AT when AS was indicated; involving the patient in the treatment decision without making a recommendation for either AS or AT; and no cancer treatment recorded. If, for example, a urologist both recommended AS and involved the patient, then their response would be marked (depending on the case) as either preferred or suboptimal treatment.

Analyses used difference-in-difference estimations for intervention versus control in choosing the preferred treatment pathway. Multinomial multivariate regressions modeled choosing the preferred treatment pathways versus each of the other three possibilities for this variable (suboptimal, indeterminate, or no treatment). We included variables for round and the intervention arm. The interaction terms of round and arm measure the differential change in the odds ratio of a physician ordering the preferred treatment (compared to suboptimal treatment) after the intervention compared to Round 1. Physician and practice characteristics were included as control variables, consisting of: CPV case, physician age, prostate cancer patient load, overall urology patient load, in-practice access to robotic surgery capability, and proportion of patients covered by Medicare and Medicaid.

We also examined the impact of protein-based assay on the overall rates of AT, both in cases where surveillance was the preferred management pathway or where treatment was preferred. This analysis provided an additional perspective of the protein-based assay’s clinical utility and role in modifying treatment patterns in clinical use.

Analyses were performed in Stata 13.0 (College Station, TX). The sample size of physicians was sufficient to detect a difference of 10% with an alpha of 0.05 and a beta of 0.80.

## Results

### Physician and practice characteristics

Participating urologists were typically in single specialty (86.3%), physician-owned (89.3%) group practices (85.9%). More than three-quarters (75.6%) have 11 or more years in practice post-fellowship and more than half (55.5%) have onsite robotic surgical capability. When looking at payor mix, these providers care for the same percentage of public versus private insurance. There were no significant differences between the two groups except a greater proportion of the intervention group reported seeing over 20 prostate cancer patients in a week (68.6%) versus the control group (46.8%) (Table 3).

**Table 3:** Baseline Physician and Practice Characteristics

	Overall	Intervention	Control	Int vs, Control *p*-value
	*n* = 129	*n* = 67	*n* = 62	
Age (Average / SD)	50.1 (8.9)	49.0 (8.9)	51.2 (8.9)	0.133
Number of years post-fellowship				
0–5	7.2%	10.2%	3.8%	0.410
6–10	17.3%	18.1%	16.3%	
11–20	45.1%	47.5%	42.3%	
21+ years	30.5%	24.1%	37.7%	
Number of MD’s associated with practice				
1–3	33.9%	34.8%	32.8%	0.816
4–10	35.5%	33.9%	37.4%	
10+	30.6%	31.3%	29.8%	
Single Specialty Practice (%)	86.3%	85.7%	87.0%	
Practice type (% breakdown)				
Group/Staff	85.9%	82.1%	90.2%	0.410
IPA	4.6%	4.8%	4.3%	
Mixed	7.9%	10.2%	5.4%	
Network	1.5%	2.9%	0.0%	
Practice Ownership (% breakdown)				
Physician-Physician group	89.3%	93.1%	85.1%	0.246
Hospital	6.3%	4.1%	8.9%	
Community Health Center	3.4%	1.4%	5.7%	
Other	0.9%	1.4%	0.3%	
Employed by practice (% Yes)	65.3%	66.7%	63.8%	0.713
Average days worked per week (%)				
4	11.0%	14.3%	7.2%	0.568
5+	89.0%	85.7%	92.8%	
On-site robotic surgery capability (%)	55.5%	51.9%	59.4%	0.364
Number of urology patients seen in 1 week				
< 50	1.6%	1.4%	1.9%	0.861
51–100	49.6%	48.9%	50.4%	
> 100	48.7%	49.6%	47.7%	
Number of prostate cancer patients seen in 1 week				
0–20	58.3%	47.3%	70.8%	0.005
> 20	41.7%	52.7%	29.2%	
Proportion of all patients covered by (sd)				
Medicare	47.6 (11.6)	48.4 (11.8)	46.7 (11.5)	0.379
Commercial	41.3(13.3)	40.3 (13.0)	42.5 (13.6)	0.316
Medicaid	6.1(6.4)	6.1 (5.9)	6.2 (6.9)	0.966
Self-pay	3.7 (4.0)	3.7 (3.8)	3.7 (4.3)	0.989

We evaluated the physicians’ AS or AT treatment choice for each case. The choices recorded in the CPVs were categorized as 1) either having appropriately recommended either AS or AT based upon guidelines for that case, 2) incorrectly choosing, 3) presenting both options equally (AS and AT) to the patients (shared decision-making), or 4) no treatment specified (no AS, AT, or shared decision-making recommended). At baseline, we found that 19.7% of the participants chose the preferred treatment, 26.0% the suboptimal treatment, 23.6% left the choice to the patient, and 30.5% did not recommend one treatment or the other, with a nonsignificant difference between control and intervention physicians (*p* = 0.645). We observed that those urologists who did not specify either AS or AT treatment were more likely to misdiagnose the patient (*p* < 0.001) or not order a biopsy (*p* < 0.001) (data not shown).

### Treatment recommendations

In bivariate analyses across all nine cases, after the protein-based array was introduced in the intervention group, the optimal treatment was selected 29.1% by the intervention arm versus 21.6% for controls (Table 4). Intervention urologists saw a 6.9% greater increase in correct treatment than the controls (*p* = 0.001) over the two rounds of data collection. Similarly, suboptimal treatment choice declined by 10.8% in the intervention group, compared to the controls (*p* = 0.028). The percentage of urologists recommending that the patient choose, decreased in the intervention group by 4.3%.

**Table 4:** Treatment Mode by Study Arm and Round (%)

	Correct Treatment		Incorrect Treatment		MDs asks patient preference (PP) (%)		No prostate cancer treatment (NT) (%)	
*Overall*	Cont.	Interv.	Cont.	Interv.	Cont.	Interv.	Cont.	Interv.
*Round 1*	17.9%	21.6%	22.4%	29.1%	25.5%	22.0%	34.2%	27.3%
*Round 2*	18.5%	29.1%	25.3%	21.1%	40.4%	32.7%	15.7%	17.1%
*D in D estimation*	6.9%		−10.8%		−4.3%		8.2%	
*p-value*	0.001		0.028		0.210		0.021	

In multinomial logistic regression, compared to controls, urologists in the intervention group were significantly more likely to recommend the preferred treatment (AS or AT) in Round 2, with an odds ratio of 2.84 (95% CI 1.39, 5.82) (*p* = 0.004). The multinomial logit accounts for the four different recommendations (preferred versus suboptimal versus patient choice versus no treatment recommendation), controlling for the individual case types and other variables of interest (Table 5).

**Table 5:** Multinomial logistic regression analysis by treatment category

	Correct Treatment			Physician counsels patient on all treatment options			No Prostate Cancer Treatment		
	Odds ratio	95% Confidence Interval	*P*-value	Odds ratio	95% Confidence Interval	*P*-value	Odds ratio	95% Confidence Interval	*P*-value
*Intervention*	0.94	(0.47,1.82)	0.860	0.57	(0.338,1.00)	0.050	0.99	(0.56,1.72)	0.960
*Interaction Round with Study Arm*	2.84	(1.39,5.82)	0.004	2.72	(1.47, 5.05)	0.001	0.98	(0.50,1.89)	0.946
*Onsite robotics capacity*	0.87	(0.51,1.49)	0.608	0.92	(0.59,1.44)	0.716	0.78	(0.49,1.26)	0.318
*More than 20 prostate cancer patients per week*	0.64	(0.35,1.15)	0.138	0.43	(0.26,0.70)	0.001	0.56	(0.33,0.95)	0.031
*More than 100 urology patients per week*	1.14	(0.64,2.03)	0.652	1.10	(0.69,1.78)	0.682	1.36	(0.81,2.26)	0.243
*Greater than 50% public payors*	0.99	(0.97,1.01)	0.400	1.00	(0.98,1.02)	0.889	1.00	(0.98,1.02)	0.768
*Age over 40*	1.02	(0.99,1.05)	0.217	1.01	(0.98,1.04)	0.435	1.05	(1.02,1.08)	0.000
*Physician-owned practice*	1.21	(0.49,2.95)	0.678	0.82	(0.41,1.64)	0.572	1.20	(0.55,2.64)	0.648
*Constant*	0.56	(0.05,6.03)	0.636	4.27	(0.63,28.78)	0.136	0.38	(0.05,2.92)	0.354

Incorrect treatment is the baseline treatment mode. Model also controls for CPV case type

The same model found that the introduction of a protein-based assay prompted urologists to involve their patients in treatment discussions more often than controls (OR 2.72; 95%CI 1.47, 5.05) (*p* = 0.001). Urologists treating more than 20 prostate cancer patients in a week were less likely to involve their patients in deciding the treatment for their cancer (*p* = 0.001).

### Changes in use of active surveillance and treatment

**Fig. 2:**
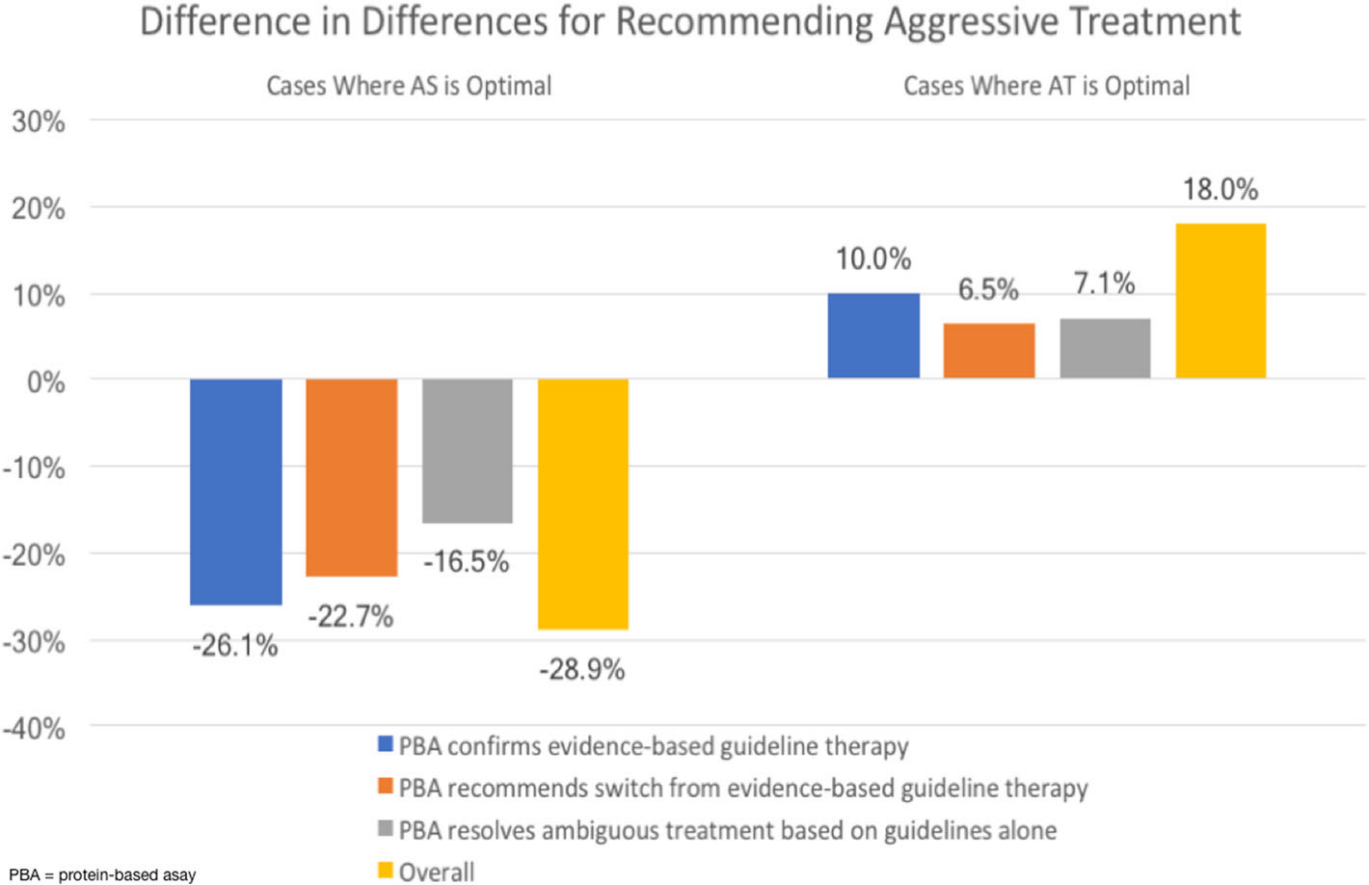
Change in AT Recommendations across Rounds, by AS and AT Cases

We specifically evaluated urologists who made either an AS or AT recommendation in Round 1 (excluding those who would counsel their patients or those who did not provide a treatment plan), grouping the cases by whether a preferred AS (6 of the 9 cases developed) or AT (3 cases) strategy was preferable. Overall, for those six cases where AS is the preferable treatment, the percentage of providers recommending AT decreased by 28.9% more in the intervention group compared to controls (Fig. 2). This decrease is observed across all three case types.

For the 3 cases where AT was preferred, urologists in the intervention group increased the recommendation for AT by 18% in Round 2 compared to controls.

## Discussion

As understanding of prostate cancer disease progression increases, the evidence indicates over-treatment with many men undergoing unnecessary prostatectomies [4]. The addition of a diagnostic tool to assess disease aggressiveness empowers clinicians to make more informed decisions on the recommended course of treatment.

This study confirms that care of prostate cancer patients, similar to many other disease states, is highly variable [18, 19]. Despite the variability in care, the results show that a protein-based assay had a positive impact on physician practice, improving treatment domain scores, and more specifically, moving patients from suboptimal to preferred recommendations, thereby providing the right treatment recommendation for the right patient.

Physicians provided with protein-based assay results changed their practice from “no recommendation” and “suboptimal recommendation” categories to either the preferable treatment pathways or engaging the patient in a conversation about their options. Importantly, this movement from the suboptimal to preferred recommendation categories by the intervention physicians maintained significance in the difference-in-differences model.

Limitations exist in this study. Since physicians responded to open-ended questions, we could not explore the counseling process for those who would present both AS and AT options to their patients. Thus, we could not determine their ultimate course of action. Likewise, a proportion of urologists did not identify the case as one of prostate cancer, and as a result, they recommended neither active treatment nor surveillance. We cannot know from these cases their treatment plan if they were presented with a prostate cancer patient.

## Conclusions

A protein-based assay provided physicians with additional prognostic information that resulted in a change to more appropriate recommendations for management, particularly in those cases where biopsy findings did not indicate a clear and precise treatment pathway. The additional information encouraged physicians to change their treatment plans. Thus, this protein-based assay shows demonstrable clinical utility, confirmed through a rigorous randomized controlled study design and regression analyses testing for effects.

## Abbreviations

AS: Active Surveillance
AT: Active Treatment
CPV: Clinical Performance and Value
